# A senescence-based prognostic gene signature for colorectal cancer and identification of the role of SPP1-positive macrophages in tumor senescence

**DOI:** 10.3389/fimmu.2023.1175490

**Published:** 2023-04-06

**Authors:** Sifei Yu, Mengdi Chen, Lili Xu, Enqiang Mao, Silei Sun

**Affiliations:** ^1^ Department of Emergency, Ruijin Hospital, Shanghai Jiao Tong University School of Medicine, Shanghai, China; ^2^ Department of General Surgery, Ruijin Hospital, Shanghai Jiao Tong University School of Medicine, Shanghai, China; ^3^ Shanghai Key Laboratory of Gastric Neoplasms, Shanghai Institute of Digestive Surgery, Ruijin Hospital, Shanghai Jiao Tong University School of Medicine, Shanghai, China

**Keywords:** colorectal cancer, senescence, prognostic model, immune infiltration, biomarkers

## Abstract

**Background:**

Senescence is significantly associated with cancer prognosis. This study aimed to construct a senescence-related prognostic model for colorectal cancer (CRC) and to investigate the influence of senescence on the tumor microenvironment.

**Methods:**

Transcriptome and clinical data of CRC cases were downloaded from The Cancer Genome Atlas (TCGA) and Gene Expression Omnibus (GEO) databases. Senescence-related prognostic genes detected by univariate Cox regression were included in Least Absolute Shrinkage and Selection Operator (LASSO) analysis to construct a model. The efficacy of the model was validated using the receiver operating characteristic (ROC) curve and survival analysis. Differentially expressed genes (DEGs) were identified and Gene Ontology (GO) and Kyoto Encyclopedia of Genes and Genomes (KEGG) pathway enrichment were performed. CIBERSORT and Immuno-Oncology Biological Research (IOBR) were used to investigate the features of the tumor microenvironment. Single-cell RNA-seq data were used to investigate the expression levels of model genes in various cell types. Immunofluorescence staining for p21, SPP1, and CD68 was performed with human colon tissues.

**Results:**

A seven-gene (PTGER2, FGF2, IGFBP3, ANGPTL4, DKK1, WNT16 and SPP1) model was finally constructed. Patients were classified as high- or low-risk using the median score as the threshold. The area under the ROC curve (AUC) for the 1-, 2-, and 3-year disease-specific survival (DSS) were 0.731, 0.651, and 0.643, respectively. Survival analysis showed a better 5-year DSS in low-risk patients in the construction and validation cohorts. GO and KEGG analyses revealed that DEGs were enriched in extracellular matrix (ECM)-receptor interactions, focal adhesion, and protein digestion and absorption. CIBERSORT and IOBR analyses revealed an abundance of macrophages and an immunosuppressive environment in the high-risk subgroup. Low-risk patients had higher response rates to immunotherapy than high-risk patients. ScRNA-seq data revealed high expression of SPP1 in a subset of macrophages with strong senescence-associated secretory phenotype (SASP) features. Using CRC tumor tissues, we discovered that SPP1^+^ macrophages were surrounded by a large number of senescent tumor cells in high-grade tumors.

**Conclusion:**

Our study presents a novel model based on senescence-related genes that can identify CRC patients with a poor prognosis and an immunosuppressive tumor microenvironment. SPP1^+^ macrophages may correlate with cell senescence leading to poor prognosis.

## Introduction

Colorectal cancer (CRC) is one of the most prevalent malignancies and the third leading cause of cancer-related mortality worldwide. Early stage CRC can be treated with surgical resection, adjuvant radiation, or chemotherapy. The standard treatment strategy for metastatic CRC is combined chemotherapy and targeted agents, including immune checkpoint inhibitors (ICIs); however, the 5-year overall survival (OS) rate remains relatively low (10–14%) in these cases (1, 2) due to drug resistance. Thus, a further understanding of the mechanisms of treatment failure in CRC remains crucial for improving the survival outcomes of CRC patients.

In the past few years, the role of senescence in cancer has been widely investigated. Cellular senescence is characterized by aberrant changes in cell morphology, gene expression, chromatin, and metabolism induced by continuous microenvironmental stimulation. Generally, cellular senescence serves as a complement to programmed cell death and helps maintain tissue homeostasis. However, the effects of senescence on cancer cells are complex. Despite their protective role in certain contexts, senescent cells may promote tumorigenesis, development, and relapse (3, 4). The senescence-associated secretory phenotype (SASP), characterized by the secretion of a series of proinflammatory chemokines and cytokines, can mediate the function of neighboring cells, such as immune cells, stromal cells, and adjacent non-tumor epithelial cells in the surrounding tumor microenvironment (TME) (5).

Recent evidence suggests the role of cellular senescence in tumor immune escape. Pereira et al. revealed that senescent cells can evade immune clearance by secreting SASP factors, such as IL-6, to upregulate HLA-E, which suppresses natural killer (NK) cells and T cell clearance in premalignant lesions (6). During the development of hepatocellular carcinoma (HCC), chemokine CCL2, another SASP factor, recruits immature suppressive myeloid cells that inhibit NK cell function and promotes the progression of HCC (7). Cellular senescence can induce drug resistance during cancer treatment. In addition, the SASP-related factor amphiregulin contributes to chemoresistance *via* upregulating programmed cell death 1 ligand (PD‐L1) expression in recipient cancer cells and creating an immunosuppressive TME (8). In summary, cell senescence can cause therapeutic resistance and lead to poor survival outcomes in cancer patients. Given the importance of cell senescence in tumors, many studies have investigated the expression of senescence-associated genes in cancer and have constructed survival prediction models. However, little is known regarding the prognostic role of senescence and its immune-mediated functions in CRC.

Bulk transcriptomics allow scientists to comprehensively understand tumor features. Thus, several analyses regarding cell senescence have been performed based on bulk transcriptome data, and attempts have been made to decompose the bulk data into lineage-specific constituents using deconvolution algorithms. However, single-cell RNA sequencing (scRNA-seq) enables the accurate identification of different cell types and recognizes their distinct characteristics in various biological states and conditions (9, 10). In the field of cell senescence, scRNA-seq has been used to understand aging of the nervous, hemopoietic, and immune systems. Thus, combined bulk transcriptome and scRNA-seq analyses provide unique insights into the SASP features of CRC and help identify potential therapeutic markers.

In this study, we constructed a senescence-related prognostic model for CRC patients based on the SenMayo gene list (11). We discovered that high-risk patients not only had poor prognosis and strong senescent features but also presented an immunosuppressive TME and resistance to immunotherapy. ScRNA-seq analysis revealed that one of the model genes, secreted phosphoprotein 1 (SPP1), was highly expressed in a subset of macrophages. This subset secretes relatively high levels of SASP factors and may contribute to the senescence of tumor cells.

## Materials and methods

### Data collection of bulk transcriptome and senescence gene sets

For the construction cohort, clinical features, RNA-seq expression data, and somatic mutation data were downloaded from the Cancer Genome Atlas-Colon Adenocarcinoma (TCGA-COAD) database (https://cancergenome.nih.gov/). For validation, clinical features and RNA-seq expression data of GSE17536, GSE17537 (12) and GSE38832 (13) were obtained from the Gene Expression Omnibus (GEO) database (https://www.ncbi.nlm.nih.gov/geo/). GSE17536 and GSE17537 were merged into a single cohort because they were derived from the same study. RNA-seq expression data of patients from the GSE213331 cohort (13) and their pathological response to neoadjuvant ICI were collected to validate the model’s ability to predict immunotherapy response. The SenMayo gene list was downloaded from the **Supplemental Material** of the study by Saul (11). Patients with unrecorded expression of genes in the SenMayo gene set were excluded.

### Construction and validation of the prognostic senescence-related gene model

Univariate Cox regression analysis was performed to identify genes predicting disease-specific survival (DSS)-predicted genes. DSS was defined as the interval from diagnosis to CRC-associated death. Senescence-related genes with |hazard ratio (HR)| > 1.0 and p-value< 0.05 were included in the model construction. To minimize the risk of overfitting, the least absolute shrinkage and selection operator (LASSO) algorithm was performed with tenfold cross validation and run for 1,000 cycles with a random stimulation of 1,000 times. Risk scores were calculated using the R package ‘glmnet.’ The senescence risk score for each patient was calculated as follows:


$$f\left(x\right)=\sum_{n=1}^{n}\left(regression\;coefficient*\;expression\;level\;of\;the\;gene\right)$$

The 1-, 2-, and 3-year ROC curves of the risk model were constructed to evaluate its prognostic performance. Patients were stratified into low- and high-risk subgroups using the median score as the cutoff value. Kaplan–Meier survival curves were plotted to compare the DSS between the two groups in the construction and validation cohorts.

### Functional enrichment analysis

We used the R package ‘limma’ to identify the expression of differentially expressed gene (DEG) sets between the high- and low-risk groups. The thresholds were set at |log2FC| > 1.0, along with a p-value< 0.05. The R package ‘clusterProfiler’ was used to explore the biological attributes of the DEGs. Kyoto Encyclopedia of Genes and Genomes (KEGG) pathway analysis, Gene Ontology (GO) pathway enrichment analysis, and Gene Set Enrichment Analysis (GSEA) were conducted. The heatmap constructed by the R package ‘ggplot2’ was used for result visualization.

### Mutation analysis

The mutation annotation format (MAF) downloaded from TCGA database was created with the ‘maftools’ package. Mutations in SenMayo genes were compared between the high- and low-risk subgroups.

### Exploration of immune-related signatures

Upregulated or downregulated immune-related pathways in the high-risk groups were analyzed using the GSEA software. We used CIBERSORT (https://cibersort.stanford.edu) to analyze the relative levels of 22 tumor-infiltrating immune cells in high- and low-risk patients. The relationship between gene expression levels and immune cell infiltration was evaluated using the TIMER2.0 database (http://timer.comp-genomics.org/). The Immuno-Oncology Biological Research (IOBR) R package was used to assess the immune features and immune cell infiltration in high- and low-risk groups.

### Single-cell sequencing data collection and processing

The raw unique molecular identifiers (UMI) count matrix of the single-cell dataset GSE132465 was downloaded (14). For quality control, the raw gene expression matrix was filtered, normalized using the Seurat R (15) package, and selected according to the following criteria: cells with > 1,000 UMI counts, > 200 genes and< 6,000 genes, and< 20% mitochondrial gene expression in UMI counts. Gene expression matrices from filtered cells were normalized and scaled. The uniform manifold approximation and projection (UMAP) method was used to lower the dimensions of the data, and t-distributed stochastic neighbor embedding (t-SNE) projection was applied to cluster and visualize the results. The cells were annotated using canonical cell surface markers. Differentially expressed genes (DEGs) were detected using the FindMarker function. Gene expression levels across various cell subtypes were determined using the DoHeatmap function.

### Immunofluorescence staining

For immunofluorescence (IF), surgical specimens of benign colon tissues (colonic diverticula), low-grade colon tumors without venous or nervous system invasion, and high-grade colon tumors with venous or nervous system invasion were collected. All procedures involving human tissue experiments were approved by the Ethics Committee of the Shanghai Ruijin Hospital, Shanghai Jiao Tong University School of Medicine, Shanghai, China. The IF staining was implemented based on formalin-fixed paraffin-embedded (FFPE) tissues which were cut into 5 µm thick slides for each panel test. The slides were dewaxed, rehydrated, and subjected to epitope retrieval by boiling in citrate antigen retrieval solution (pH = 6; Servicebio #G1206) for 3 min. After cooling, the slides were washed three times in phosphate-buffered saline (PBS) for 5 min. Proteins were blocked with bovine serum albumin (BSA) for 30 min. One antigen was added in each round, including the BSA block, primary and secondary antibody incubation, and antigen retrieval. The above procedures were repeated until the three biomarkers, CD68 (Servicebio #GB113150, 1:3000), p21 (Servicebio #GB11153, 1:4000), and SPP1 (abcam # ABS135915, 1:200), were added. The secondary antibodies for the three biomarkers were horseradish peroxidase (HRP)-labeled goat anti-rabbit antibody (Servicebio #GB23303, 1:500) for CD68 and p21 and Cy3-labling goat anti-rabbit antibody (Servicebio #GB21303, 1:500). An AutoFluo quencher was applied and the nuclei were stained with 4, 6-diamidino-2- phenylindole (DAPI, Servicebio #GB1012) before the slides were blocked using an antifade mounting medium (Servicebio #GB1401). Images were captured using an inverted fluorescence microscope.

### Statistical analysis

Statistical analyses were conducted using R software (version 4.2.2, https://www.r-project.org/) and its appropriate packages. Kaplan–Meier analysis was used to assess and compare survival between the different subgroups. Log-rank two-tailed p< 0.05 was considered as statistically significant.

## Results

### Construction of senescence-related model

**Figure 1:**
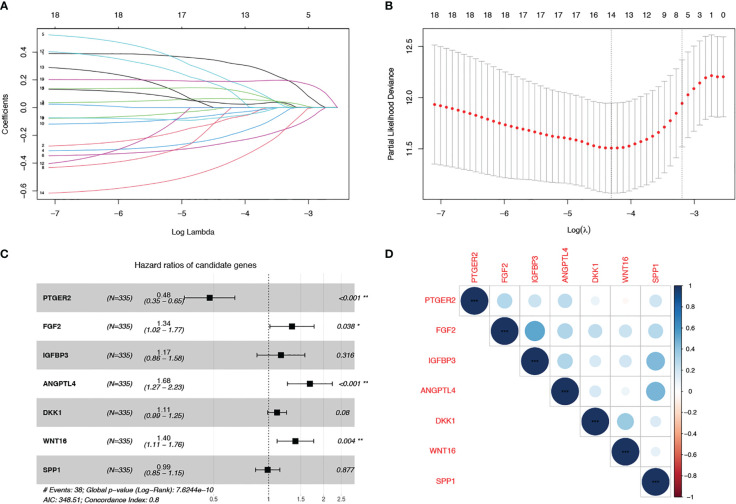
Construction of a cell senescence-associated prognostic model based on the SenMayo gene set. **(A, B)** LASSO Cox regression analysis was conducted to screen the key genes; **(C)** Forest plots showing the results of the univariate Cox regression analysis between overlapping genes and overall survival; **(D)** Correlation of the seven model gene expression.

The whole work diagram was summarized in **Supplemental Figure 1**. Among the 125 SenMayo genes, 18 were prognostic according to univariate Cox regression analysis. LASSO Cox regression analysis was conducted to build a multigene prognostic model with the least chance of overfitting, based on the 18 genes (**Figures 1A, B**). Seven genes were finally selected into the model according to the optimal value of λ and the multivariate hazard ratios are shown in **Figure 1C**. The genes included prostaglandin E receptor 2 (PTGER2), fibroblast growth factor 2 (FGF2), insulin like growth factor binding protein 3 (IGFBP3), angiopoietin like 4 (ANGPTL4), dickkopf WNT signaling pathway inhibitor 1 (DKK1) and wingless-type MMTV integration site family member 16 (WNT16). The risk score was calculated using the following formula: senescence risk score = (-0.744 × expression level of PTGER2) + (0.295 ×the expression level of FGF2) + (0.155 × the expression level of IGFBP3) + (0.520 × the expression level of ANGPTL4) + (0.106 × the expression level of DKK1) + (0.337 × the expression level of WNT16) + (0.012 × expression level of SPP1). The correlation matrix (**Figure 1D**) revealed that the expression of each model gene was independent of other genes.

### Validation of the model in the training and validation cohorts

**Figure 2:**
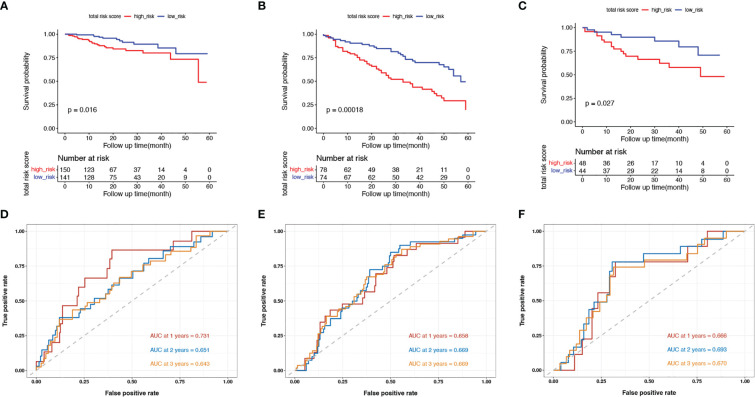
Validation of the model in the training cohort and validation cohorts. **(A–C)** Kaplan-Meier survival curves of in senescent low- and high-risk patients in the TCGA training cohort **(A)**, the GSE17536 and GSE17537 validation cohort **(B)** and the GSE38832 validation cohort **(C)**; **(D–F)** The ROC curves at 1-, 2- and 3-year in the mentioned three cohorts.

Next, we evaluated the prognostic value of our model in TCGA and validation cohorts (GSE17536/7 and GSE38832). In each cohort, we categorized patients into low- and high-risk groups using the median value as the threshold. Survival analysis was performed for low- and high-risk patients. In the training cohort, the 5-year DSS rates were 84.7% (95% confidence interval (CI): 75.6–94.9%) for low-risk patients and 71.7% (95% CI: 59.0–87.1%) for high-risk patients (p = 0.016, **Figure 2A**). In the two validation cohorts, high-risk patients had worse survival outcomes than low-risk patients (p< 0.001 in GSE17536/7, **Figure 2B**; p = 0.027 in GSE38832, **Figure 2C**). The AUC at 1, 2, and 3 years were 0.731, 0.651, and 0.643, respectively, in TCGA cohort (**Figure 2D**); 0.658, 0.669, and 0.669, respectively, in GSE17536/7 cohort (**Figure 2E**); and 0.666, 0.693, and 0.670, respectively, in GSE38832 cohort (**Figure 2F**). Taken together, our senescence-related 7-gene risk model accurately distinguished high-risk patients from low-risk patients, and its prognostic ability was stable.

### Clinicopathological features of senescent high- and low-risk patients

In addition to the prognosis, we investigated the basic clinicopathological features of the high- and low-risk groups (**Table 1**). High-risk patients were more likely to have tumors with venous invasion (p = 0.001) and higher American Joint Committee on Cancer (AJCC) T stages (p = 0.006), and N stages (p = 0.001). The proportion of metastases was also higher in the high-risk patients (p = 0.012). In summary, the clinicopathological features were more aggressive in the senescent high-risk patients than in the low-risk patients.

**Table 1:** Comparison of basic clinicopathological features in high-risk and low-risk groups.

Characteristics	High risk n=167	Low risk n=168	p-value
MS type			0.497
MSI-H	29 (17.4)	21 (12.5)	
MSI-L	25 (15.0)	26 (15.5)	
MSS	109 (65.3)	114 (67.9)	
Unknown	14 (8.3)	7 (4.1)	
Venous Invasion			0.001
Yes	48 (28.7)	24 (14.3)	
No	97 (58.1)	126 (75.0)	
Unknown	22 (13.2)	18 (10.7)	
Pathologic T			0.006
T1	1 (0.5)	9 (5.3)	
T2	20 (12.0)	31 (18.5)	
T3	118 (70.7)	112 (66.7)	
T4	28 (16.8)	16 (9.5)	
Pathologic N			0.001
N0	77 (46.1)	110 (65.5)	
N1	52 (31.1)	38 (22.6)	
N2	38 (22.8)	20 (11.9)	
Pathologic M			0.012
M0	109 (65.3)	120 (71.4)	
M1	33 (19.8)	15 (8.9)	
Unknown	25 (14.9)	33 (19.6)	
Pathologic stage			<.001
I	20 (12.0)	36 (21.4)	
II	54 (32.3)	72 (42.9)	
III	58 (34.7)	45 (26.8)	
IV	35 (21.0)	15 (8.9)	

MS, microsatellite; MSI, microsatellite instability; MSS, microsatellite stability; T, tumor; N, lymph node; M, metastasis.

### The landscape of DEGs and mutations in high-risk and low-risk subgroups

**Figure 3:**
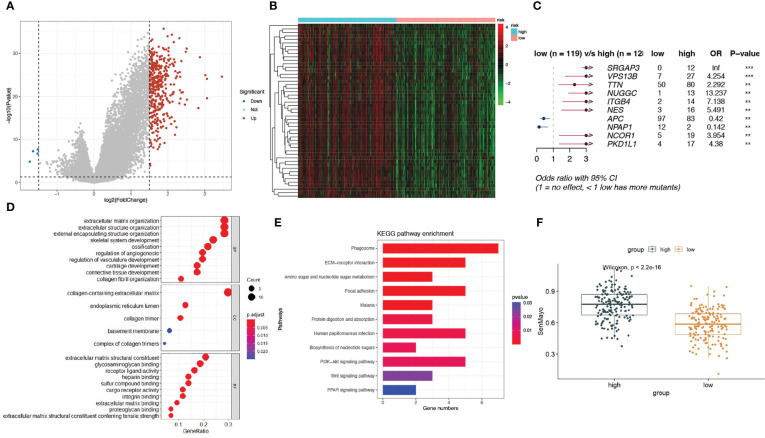
A landscape of different expressed genes and mutations in high-risk and low-risk subgroups. **(A)** Volcano plots of differentially expressed genes (DEGs) between high-risk and low-risk patients; **(B)** Heatmap of the expression of top 50 DEGs in the two subgroups; **(C)** The differentially mutated SenMayo genes between the two groups; **(D)** The GO enrichment pathways of DEGs; **(E)** The KEGG enrichment pathways of DEGs; **(F)** Comparison of SenMayo enrichment score between two subgroups.

To explore the genomic characteristics of low- and high-risk patients, we identified DEGs between the two subgroups using a fold change cutoff value of 1.5 and a p-value< 0.05 (**Figure 3A**). Compared with the low-risk group, there were 375 upregulated and 4 downregulated genes in the high-risk group. The expression of the top50 upregulated genes in high-risk patients is shown in a heatmap (**Figure 3B**). We then examined the mutation status of the SenMayo genes in the two groups and listed the top 10 differentially mutated genes. More mutated senescence-related genes, including SLIT-ROBO Rho GTPase activating protein 3 (SRGAP3), vacuolar protein sorting 13 homolog B (VPS13B), titin (TTN), nuclear GTPase, germinal center associated (NUGGC), integrin subunit beta 4 gene (ITGB4), nestin (NES), nuclear receptor corepressor 1 (NCOR1), and polycystic kidney disease protein 1-like 1 (PKD1L1), were found in the high-risk group (**Figure 3C**). GO pathway analysis revealed enrichment mainly in the extracellular matrix (ECM) and structure-related pathways (**Figure 3D**). The top 1 KEGG enrichment pathway was phagosome, which participates in the elimination of senescent cells. Others included ECM-receptor interactions, focal adhesions, and protein digestion and absorption (**Figure 3E**). We calculated the SenMayo signature scores of the two subgroups and confirmed that the scores were significantly higher in the high-risk patients (**Figure 3F**), indicating that this group was burdened with strong SASP features.

### Comparison of TME features in the high-risk and low-risk groups

**Figure 4:**
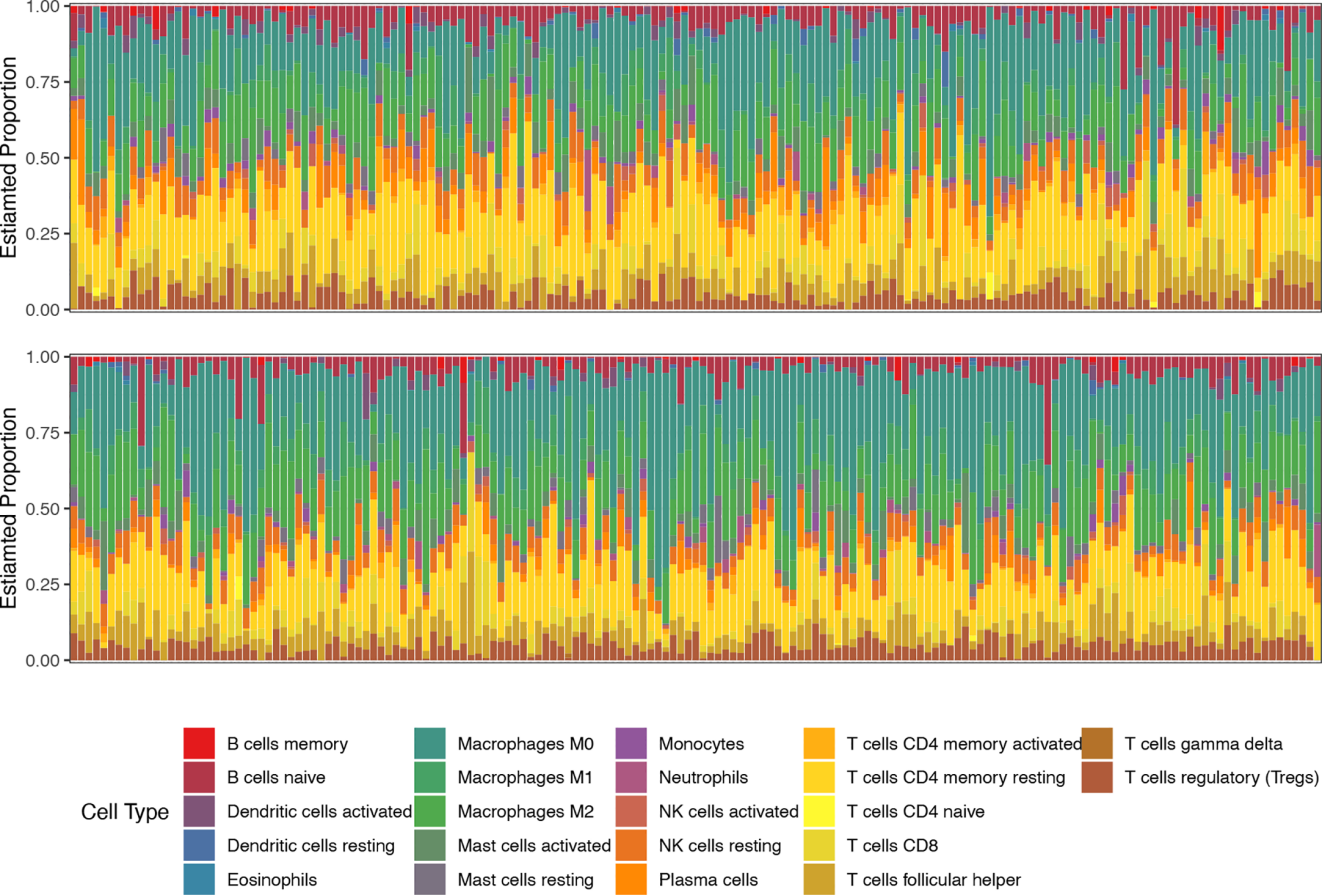
The proportions of 22 types of immune cells in each sample of low-risk (top line) and high-risk (bottom line) groups revealed by CIBERSORT.

**Figure 5:**
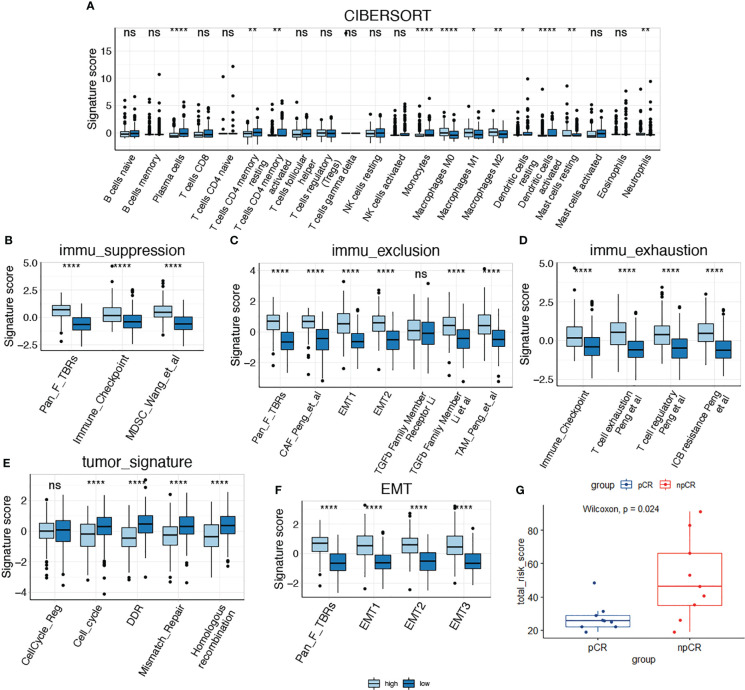
Comparison of tumor microenvironment features in high-risk and low-risk groups. **(A)** Comparison of the infiltration of immune cells between two subgroups; **(B–D)** Comparison of immune suppression, exclusion and exhaustion features between two subgroups; **(E, F)** Comparison of tumor signatures and EMT signatures between two subgroups; **(G)** Analysis of the senescence risk score in patients with different response to immunotherapy using a rectal cancer cohort (GSE213331). *P < 0.05; **P < 0.01; ****P < 0.0001; NS, not significant.

We then compared the TME features between the high- and low-risk subgroups. Using the CIBERSORT algorithm, we analyzed the abundance of 22 different immune cells in the two groups (**Figure 4**). The proportions of plasma cells, CD4^+^ memory T cells, monocytes, and dendritic cells were obviously lower in the high-risk subgroup than in the low-risk subgroup (**Figure 5A**). In contrast, the infiltration levels of macrophages (including the M1 and M2 subtypes), mast cells, and neutrophils were significantly higher in high-risk patients. Further analysis of TME signatures using the IOBR package revealed that the TME of high-risk patients was immunosuppressive, exclusive, and exhausted (**Figures 5B–D**). Moreover, low-risk patients may be more sensitive to immunotherapy according to their higher mismatch repair (MMR) and homologous recombination scores (**Figure 5E**). High-risk patients exhibited stronger epithelial-mesenchymal transition (EMT) signatures (**Figure 5F**). Collectively, these results indicate an immunosuppressive TME in high-risk patients. Therefore, we examined the association between senescence risk score and the efficacy of immunotherapy in a rectal cancer cohort. Non-pCR patients had significantly higher senescence risk scores than pCR patients (p = 0.024; **Figure 5G**). We also compared the expression other IO biomarkers in the two subgroups. While no difference of tumor mutation burden (TMB) was found, we discovered significant enhanced PD-L1 expression in the high-risk group. (**Supplemental Figure 1**)

### Identification of SPP1^+^ macrophages as a key component in cell senescence based on scRNA transcriptomic analysis

**Figure 6:**
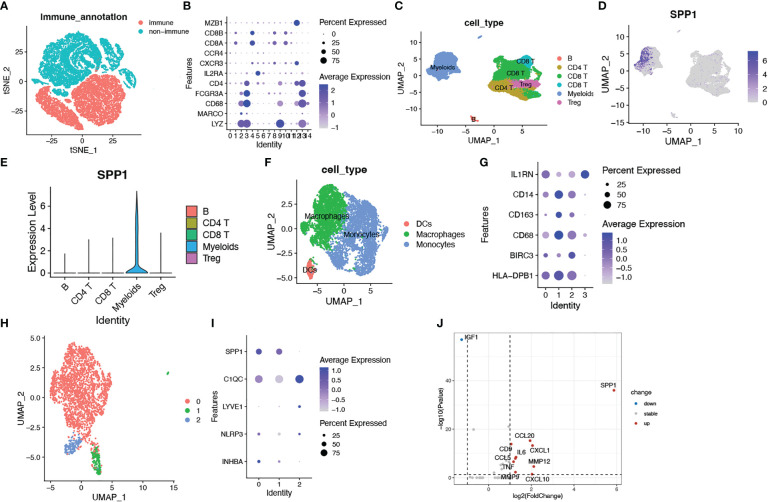
Identification of SPP1^+^ macrophages as a key imponent in cell senescence of colorectal cancer based on scRNA transcriptomic analysis. **(A)** tSNE plots showing immune cell and non-subsets identified by the CD45 marker; **(B)** Bubble plots showing the expression of marker genes in all immune cell clusters. Dot size indicates the percent expressed genes and color indicates the expression strength levels; **(C)** UMAP plot showing immune cell clusters defined according to the marker genes; **(D)** The expression of SPP1 on all immune cells; **(E)** Violin plot showing SPP1 expression on various types of immune cells; **(F)** UMAP plot showing myeloid cell clusters defined according to the marker genes; **(G)** Bubble plots showing the expression of marker genes in myeloid cell clusters; **(H)** UMAP plot showing three subsets of macrophages; **(I)** Bubble plots showing macrophage related genes; **(J)** Volcano plots showing differentially expressed SenMayo genes between SPP1^+^ and SPP1- macrophages.

**Figure 7:**
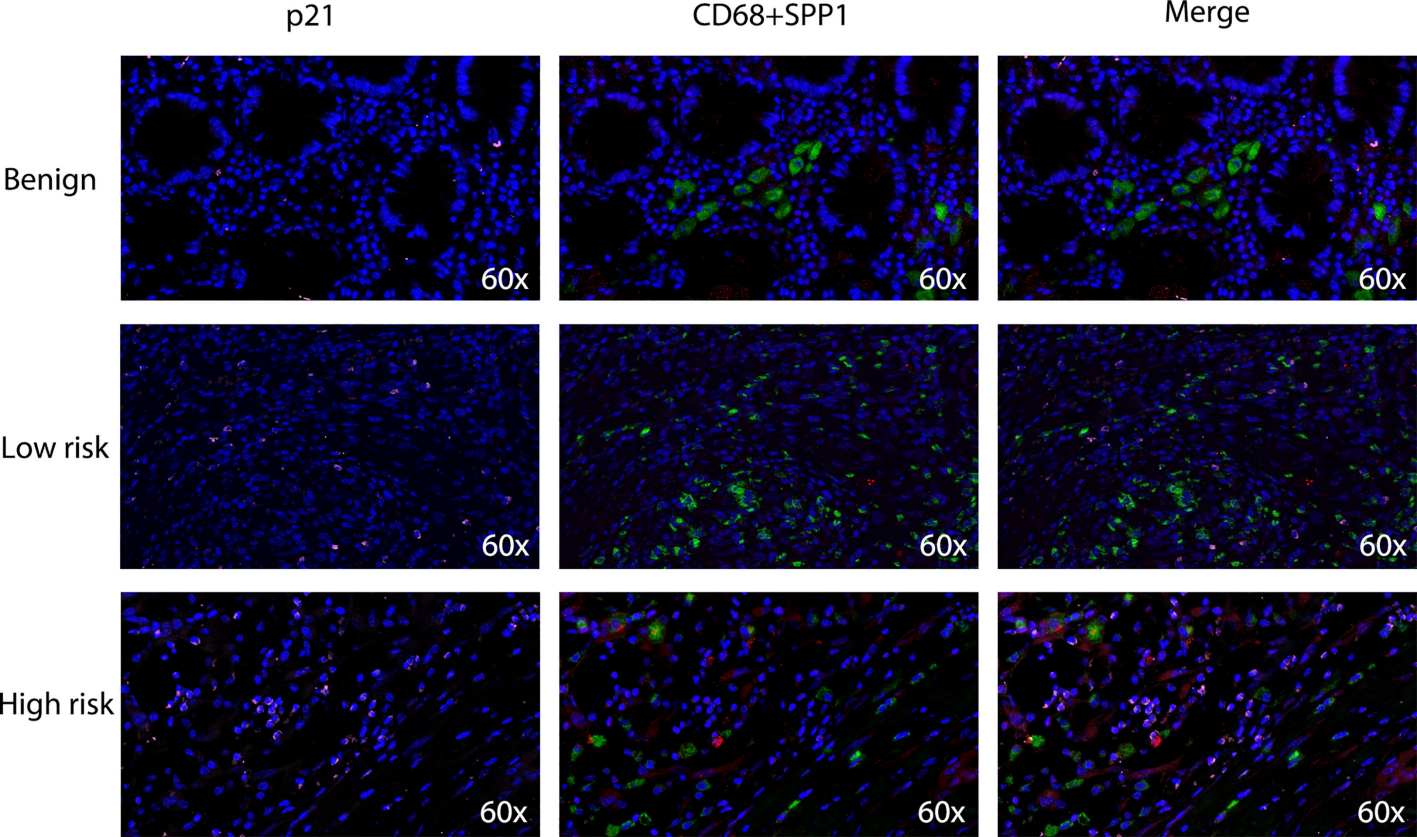
Densities of tumor-infiltrating SPP1^+^/CD68^+^ macrophages and p21^+^ senescent cells in benign colon tissues, low-grade colon tumor tissues and high-grade colon tumor tissues. Confocal microscopy scan of immunofluorescence staining showed the distribution of SPP1 (red) positive CD68 (green) double positive macrophages and p21 (pink) positive senescent cells.

Based on the close relationship between the immune microenvironment and cellular senescence, we focused on the expression of our model genes in immune cells using scRNA transcriptomic analysis. After scaling and normalizing the expression matrix, we used CD45 as a marker to broadly categorize the cells into immune and non-immune cell populations (**Figure 6A**). Immune cells were selected and dimensionality reduction was performed. Using specifical canonical markers defined in the literature, cells were divided into the following clusters (**Figures 6B, C**): B cells (‘MZB1’), CD4 positive cells (‘CD4,’ ‘IL2RA,’ ‘CXCR3,’ ‘CCR4’), CD8 positive cells (‘CD8A,’ ‘CD8B’), regulatory T cells (‘IL2RA’), and myeloid cells (‘LYZ,’ ‘MARCO,’ ‘CD68,’ ‘FCGR3A’). The expression levels of the model genes were examined in the five populations (**Supplementary Figure 2**). Strong expression of SPP1 was observed, particularly in myeloid cells (**Figures 6D, E**). Given the significantly enhanced infiltration of macrophages in high-risk tumors, we selected myeloid cell clusters for further analysis. The myeloid cell population was divided into dendritic cells (DCs) (‘BIRC3,’ ‘HLA-DPB1’), macrophages (‘CD163,’ ‘CD68,’ ‘CD14’) and monocytes (‘IL1RN’) as shown in **Figures 6F, G**. After clustering the macrophages into three subgroups by dimensionality reduction, we found that SPP1 was highly expressed, particularly in cluster 0 (**Figures 6H, I**). We compared the expression of the SenMayo genes between clusters 0 and 1. In addition to SPP1, nine other senescence-related genes were upregulated in cluster 0 macrophages, indicating the SASP features of this sub-cluster (**Figure 6J**). We performed immunofluorescence assays to detect the association between SPP1^+^ macrophages and tumor senescence. Normal colon tissues, low-grade colon tumor tissues, and high-grade colon tumor tissues were stained. A larger number of SPP1 (red)-positive macrophages (green) and surrounding senescent tumor cells (p21 positive, pink) were observed in high-grade tumors than in low-grade tumors. In normal tissues, the proportions of SPP1^+^ macrophages and p21^+^ tumor cells were low (**Figure 7**).

## Discussion

CRC is one of the most prevalent malignancies with high mortality rates worldwide. In this study, we constructed a senescence prognostic model based on the SenMayo gene panel using public bulk transcriptome data and discovered a relationship between SASP and the immunosuppressive microenvironment. We investigated the expression of senescence prognostic genes in scRNA-identified immune cell populations and identified SPP1^+^ macrophages as an important TME component that leads to tumor senescence.

Cellular senescence is elicited by various intrinsic and extrinsic stresses, including replicative exhaustion and cancer therapies such as chemotherapy and radiation. SenMayo is a novel gene set designed by Saul et al. to identify cells expressing high levels of SASP genes and to evaluate the clinical senescence burden. Based on SenMayo, we constructed a prognostic model that could distinguish CRC patients with strong SASP features and poor survival outcomes.

The model consisted of the following seven genes: PTGER2, FGF2, IGFBP3, ANGPTL4, DKK1, WNT16, and SPP1. Among these risk factors, FGF2 (also known as bFGF, a basic fibroblast growth factor) is a well-known survival factor, and a higher level of FGF2 is secreted by senescent cells than by pre-senescent cells. It has also been reported that FGF2 can shift macrophages towards an M2-like phenotype and alter tumor immunity, which can therefore be a therapeutic target in cancer treatment (16). IGFBP3 is known for its pleiotropic ability to regulate cell proliferation, apoptosis, and differentiation. It has recently been shown that IGFBP3 is an upregulated secretory factor of senescent cells and is associated with SASP (16–18). ANGPTL4 encodes a secreted glycoprotein that promotes angiogenesis and inhibits ferroptosis (19). ANGPTL4 participates in tumorigenesis and therapeutic resistance through autocrine and paracrine activity (20–23). DKK1 is a WNT signaling pathway inhibitor that could trigger early onset of the cellular senescence (24, 25). WNT16 is a secreted signaling protein that is overexpressed during stress- and oncogene-induced senescence (26). A previous study has reported that paracrine WNT16B attenuates the effects of cytotoxic therapy (27). SPP1 is a secreted cytokine closely associated with tumorigenesis, invasion, and metastasis. SPP1 could upregulate the expression of interferon (IFN)-γ and interleukin (IL)-12 and modulate the function of various TME components. Importantly, previous studies have reported that a special subtype of tumor-associated macrophages (TAM) with strong SPP1 expression presents immunosuppressive features and is positively correlated with EMT markers (28–30).

As cell senescence can modulate the immune environment, we further investigated the immune features of senescent high-risk patients. Immune-related gene signature sets indicate an immunosuppressive phenotype in senescent high-risk tumors. According to the results of CIBERSORT, this population distinctly exhibited highly infiltrating macrophages. Thus, we hypothesized that macrophages contribute to tumor cell senescence and SASP features in high-risk patients.

To evaluate the expression of senescence-related genes in immune cells, we identified immune cell populations using scRNA-seq data and further divided them into various subtypes. We found a particularly high expression of SPP1, one of our model genes, in myeloid cells. Based on previous evidence and our CIBERSORT results, we next focused on the expression of SPP1 in macrophages. It has been discovered that there are two distinct subsets of TAMs in CRC, the SPP1^+^ subset and the C1QC^+^ subset. While C1QC^+^ TAMs preferentially express phagocytosis- and antigen presentation-related genes, SPP1^+^ TAMs have a proangiogenic signature and are more likely to engage in crosstalk with cancer-associated fibroblasts (CAFs) and endothelial cells (29, 31). Patients with strong SPP1^+^ TAM infiltration show resistance to immunotherapy and poor prognosis. Our study is the first to report that SPP1^+^ TAMs exhibit stronger SASP features than C1QC^+^ TAMs. This subpopulation of TAMs highly expresses senescent factors such as CCL20, CXCL1, MMP12, CXCL10, IL6, and CCL5. Using human benign colon tissues and colon tumor tissues, we found that SPP1^+^ macrophages were particularly enriched in high-grade tumors. We observed a large number of senescent tumor cells around the SPP1^+^ macrophages, whereas there were fewer SPP1^+^ macrophages and senescent cells in low-grade tumors and benign colon tissues. This result further indicates the role of SPP1^+^ macrophages in the development of SASP features in CRC. Therefore, targeting SPP1^+^ macrophages may alter the senescent state of tumor cells and reverse immunotherapeutic resistance.

Our study has several limitations. First, our model was based on gene expression in CRC patient samples, and the incorporation of clinical factors may have improved the efficacy of the model score. The predictability of immunotherapy response in our model needs to be further validated in larger cohorts. The intrinsic association between macrophages and the senescent tumor environment revealed by our model should be further investigated *in vivo* and *in vitro*. Despite these limitations, our study provides novel insights into senescence-immune interactions in CRC and an effective prognostic model to guide ICI treatment.

## Conclusion

Our study presents a novel model based on senescence-related genes that can identify CRC patients with a poor prognosis and an immunosuppressive TME. SPP1^+^ macrophages may correlate with cell senescence, leading to a poor prognosis.

## Data availability statement

The datasets presented in this study can be found in online repositories. The names of the repository/repositories and accession number(s) can be found within the article/**Supplementary Material**.

## Ethics statement

The studies involving human participants were reviewed and approved by Ethics Committee of Shanghai Ruijin Hospital, Shanghai Jiao Tong University School of Medicine. The patients/participants provided their written informed consent to participate in this study.

## Author contributions

EM and SS contributed to conceptualization, supervision and writing- review and editing. SY performed investigation, data duration and writing-original draft. MC performed data duration and verification. LX worked on investigation and software. All authors contributed to the article and approved the submitted version. 

## Supplementary material

Supplementary Figure 1 The work flowchart of our study.

Supplementary Figure 2 The correlation between **(A)** PD-L1 or **(B)** TMB and angiogenetic risk scores.

Supplementary Figure 3 Expression of model genes across immune cells in CRC. WNT16 were not detected in this scRNA-seq dataset.
