# Supplementary figures and images for: A senescence-based prognostic gene signature for colorectal cancer and identification of the role of SPP1-positive macrophages in tumor senescence
Source: Front Immunol. 2023 Apr 6;14:1175490. doi: 10.3389/fimmu.2023.1175490 (PMC10115976; doi:10.3389/fimmu.2023.1175490)

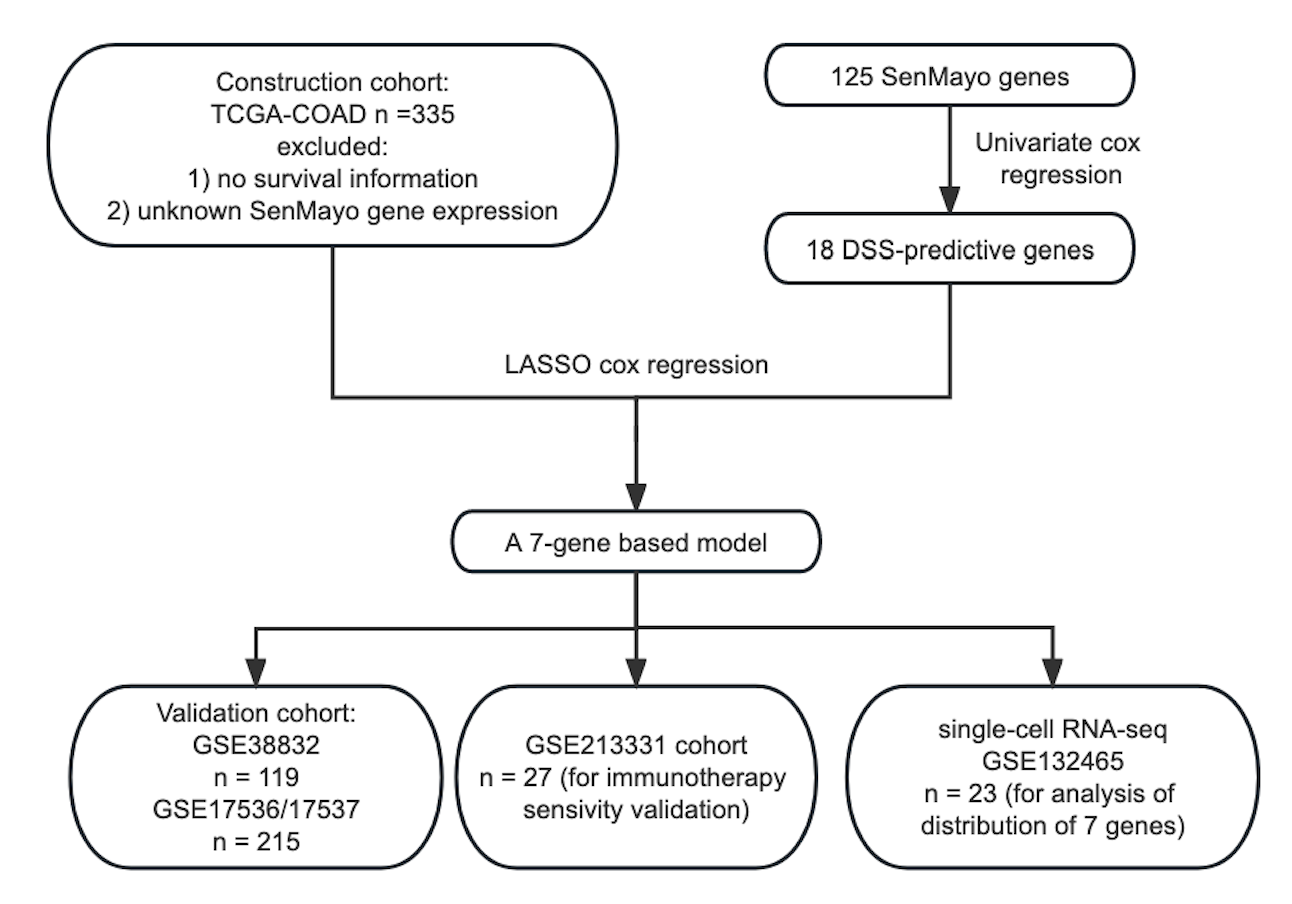

Supplement: Supplementary Figure 1 — The work flowchart of our study. [file Image_1.png]

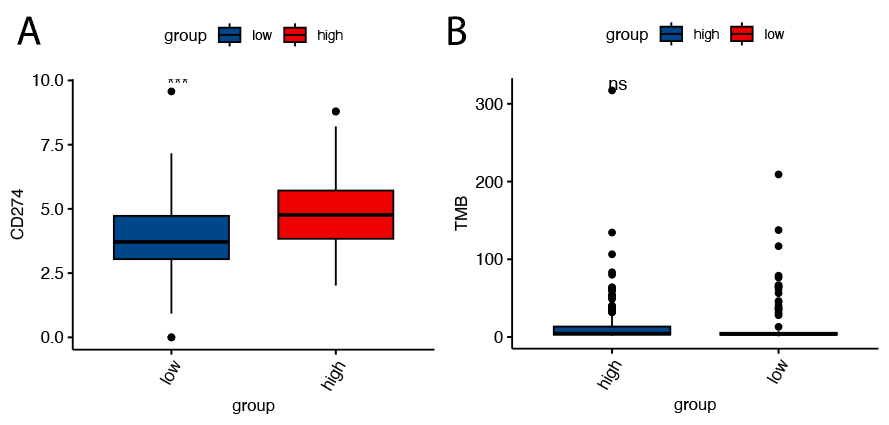

Supplement: Supplementary Figure 2 — The correlation between (A) PD-L1 or (B) TMB and angiogenetic risk scores. [file Image_2.tif]

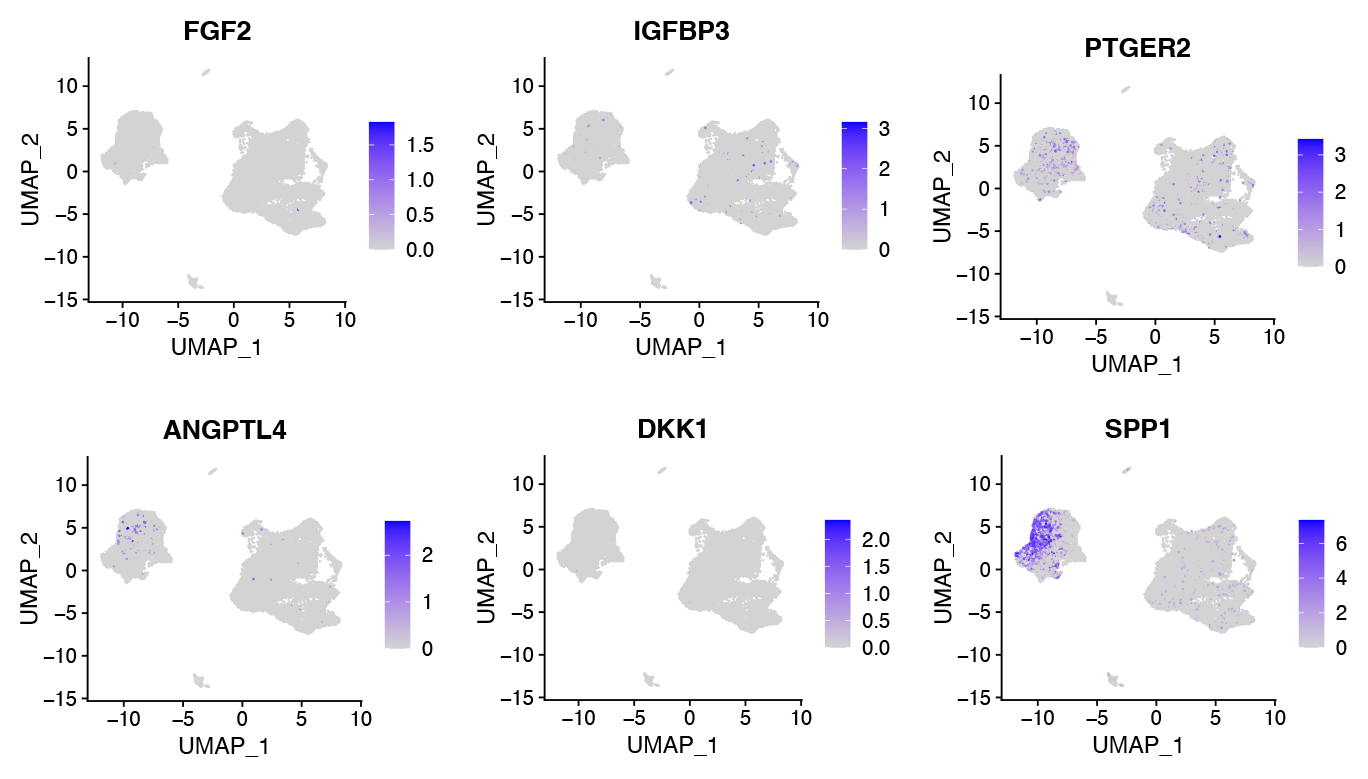

Supplement: Supplementary Figure 3 — Expression of model genes across immune cells in CRC. WNT16 were not detected in this scRNA-seq dataset. [file Image_3.tif]
